# Nodular Fasciitis—A Rare Cause of a Rapidly Growing Ear Lesion in a 19-Month-Old Child

**Published:** 2020-10-19

**Authors:** Jordan N. Halsey, Julien Hohenleitner, Frank S. Ciminello

**Affiliations:** ^a^Division of Plastic and Reconstructive Surgery, Department of Surgery, Rutgers New Jersey Medical School, Newark; ^b^Craniofacial & Pediatric Plastic & Reconstructive Surgery, Department of Plastic Surgery & Neurosurgery, Hackensack University Medical Center, Hackensack, NJ

**Keywords:** nodular fasciitis, pediatric masses, auditory canal lesion, locally destructive lesions, ear mass

## CASE DESCRIPTION

**Figure 1:**
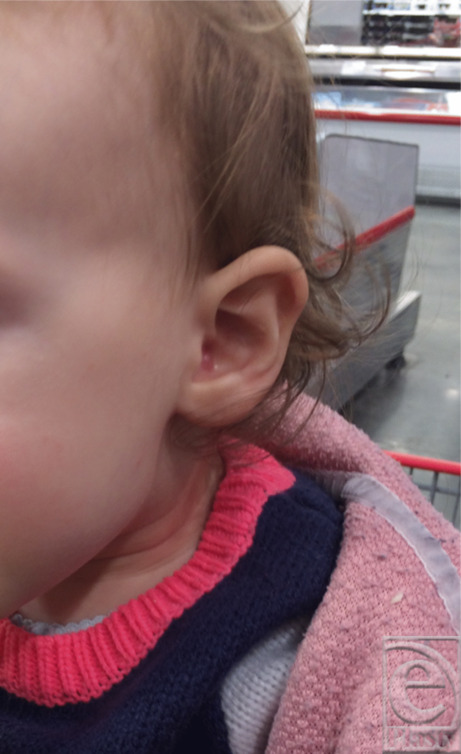
Left ear lesion at 9 months of age.

**Figure 2:**
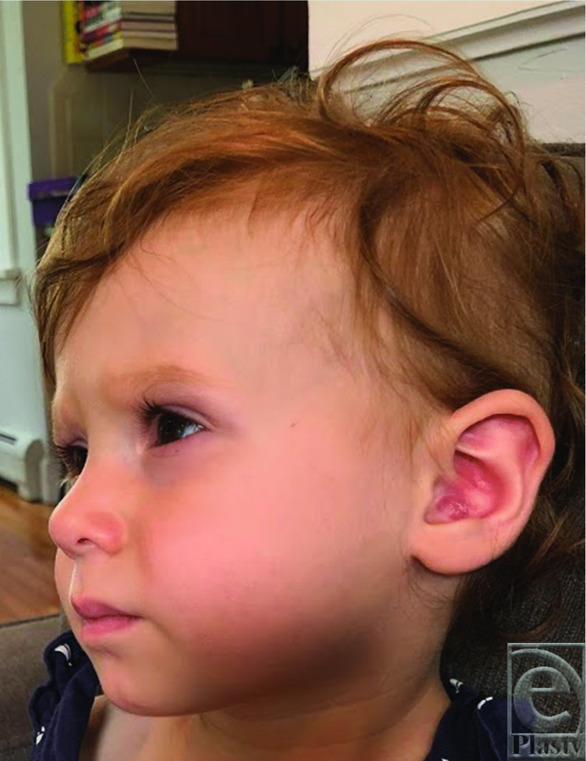
Left ear lesion at 19 months of age, prior to the period of rapid proliferation.

**Figure 3:**
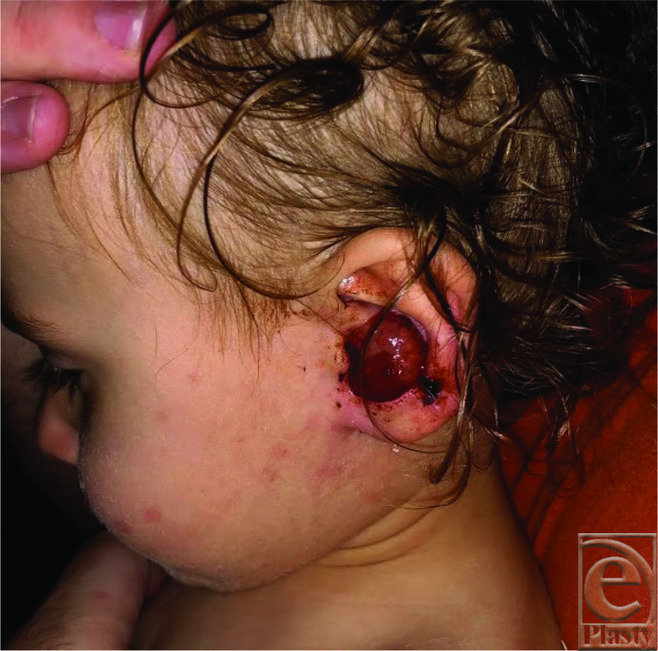
Left ear lesion in the child at 19 months of age, 3 days after mild trauma while playing at home.

A 9-month-old patient developed a small, skin-colored lesion in the conchal bowl of her left ear (Fig 1). There were no auditory abnormalities or pain associated with the lesion. The patient's dermatologist thought this mass to be a keloid and performed a steroid injection, with no treatment response. Ten months later, after minor trauma to the lesion during play at home, the lesion rapidly grew, ulcerated, and became prone to bleeding. Figure 2 shows the lesion prior to the trauma; Figure 3 shows the growth posttrauma. A biopsy was performed; the results were consistent with nodular fasciitis. The patient was referred to plastic surgery for evaluation. At the time, the lesion was 2.1 × 2.1 × 0.8 cm in size and invaded the entire conchal bowl, with near complete obstruction of the external auditory canal. An active clot with blood staining along the lobule and periauricular region was present.

## QUESTIONS

1. What is nodular fasciitis, and why is this case unique?
2. What other lesions can mimic nodular fasciitis on physical examination?
3. What are distinguishing histologic features of nodular fasciitis on pathologic examination?
4. What is the best way to treat nodular fasciitis to prevent recurrence?

## DISCUSSION

Nodular fasciitis is a benign, reactive, tumor-like proliferation of myofibroblastic and fibroblastic cells that most often affects subcutaneous tissue, muscle tissue, and fascia.^1^ The lesion was first described by Konwaler et al^2^ in 1955. It is commonly mistaken for a sarcoma or proliferative myositis due to rapid growth, high cellularity, and mitotic activity.^3^ Nodular fasciitis can develop in multiple places on the body, including the upper extremities, head, neck, and auricular regions. The cause is uncertain; however, preceding trauma is noted in 10% to 18% of cases.^4^ Gan et al^5^ reported 64 cases of nodular fasciitis, with patient ages ranging between 3 and 84 years. Of those case patients, only 3 were younger than 10 years and only 13 (20.3%) had a mass located in the head and neck. The mean age of all case patients in their study was 37.5 years. A similar study conducted by Thompson et al^6^ reported 50 cases of auricular nodular fasciitis, and only 6 of those cases (12%) were located in the external auditory canal. In all reported cases of nodular fasciitis at their institution, only 1.5% presented in the ear. Of the 50 case patients, the ages ranged from 1 to 76 years, with a mean age of 27.4 years. While reported cases do show a propensity for this lesion to develop in younger patients, there are very few instances in the literature describing this lesion in a patient younger than 2 years.

It can be difficult to differentiate nodular fasciitis from malignancy, both clinically and by imaging. Any rapidly growing solitary mass could suggest a variety of malignant lesions and lead to severe patient anxiety. Nodular fasciitis has been misdiagnosed by contributing pathologists in 75% of the cases studied by Thompson et al^6^; in 30% of those cases, the lesion was considered a sarcoma. There was an overall 23% malignant misdiagnosis in their cases. Physicians must differentiate nodular fasciitis from other benign and malignant spindle cell neoplasms such as benign fibrous histiocytoma, myxoma, inflammatory myofibroblastic tumor, or myxofibrosarcoma. While computed tomography or magnetic resonance imaging may be helpful in delineating the extent of the mass, it is often not helpful in differentiating the lesion from a malignancy or locally destructive tumor. The only way to appropriately make the pathologic diagnosis of nodular fasciitis is via histologic analysis of the specimen by the pathologist. Nodular fasciitis’ unique features, including high cellularity, dispersed cells, cell polymorphism, and myxoid background, must be recognized to distinguish it from other conditions.^7^ Overall, proper diagnosis is vital in an effort to avoid overly aggressive or invasive treatment where a much simpler treatment is often indicated.

**Figure 4:**
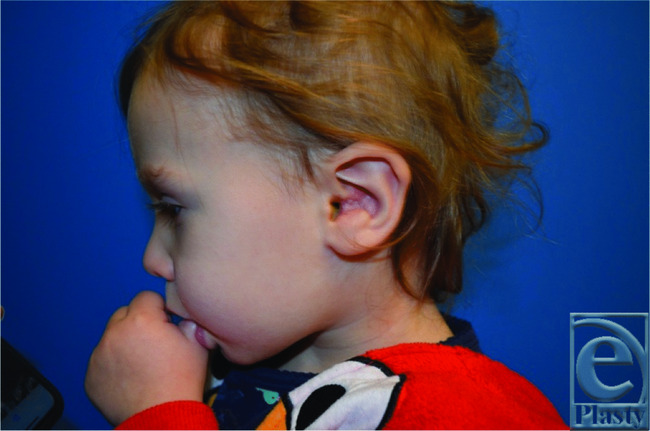
Eleven days following surgical resection. Skin graft is well healing, and the ear has an overall excellent aesthetic appearance postreconstruction.

The recommended treatment of nodular fasciitis is complete local excision.^8^ Once the lesion is fully excised, it will rarely recur. Interestingly, the majority of recurrences take place in children and most commonly in the auricular region. This is thought to be due to difficulty achieving complete margins in this particular area.^4^ In our patient, we performed an entire resection of the lesion, including margins and the underlying cartilage, with immediate reconstruction producing an aesthetically acceptable result (Fig 4). To date, there has been no sign of recurrence. To fully excise the mass, a radical excision was performed. A margin of what appeared to be normal tissue was excised in all directions including the underlying cartilage, which involved resection of the entire conchal bowl. All specimens were sent to pathology. The large auricular defect was reconstructed with a full-thickness skin graft along the region of cartilage resection and the external auditory canal. This was harvested from the patient's postauricular region, 5 × 5 cm in size. An adjacent tissue transfer using a z-plasty design was performed at the level of the external auditory canal to prevent narrowing. No cartilage reconstruction was performed, as it was unnecessary to produce an aesthetically favorable result.

In conclusion, nodular fasciitis of the external auditory canal is an exceedingly rare mass that can present both diagnostic and reconstructive challenges for the pathologist and the surgeon, respectively. Appropriate diagnosis can often be difficult to ascertain, considering its features often mimic much more serious malignant conditions. Recurrence is known to be highest in auricular nodular fasciitis; thus, complete local excision must be performed carefully to prevent recurrence.
